# Evaluation of a Blended Relapse Prevention Program for Anxiety and Depression in General Practice: Qualitative Study

**DOI:** 10.2196/23200

**Published:** 2021-02-16

**Authors:** Esther Krijnen-de Bruin, Jasmijn A Geerlings, Anna DT Muntingh, Willemijn D Scholten, Otto R Maarsingh, Annemieke van Straten, Neeltje M Batelaan, Berno van Meijel

**Affiliations:** 1 Amsterdam UMC, Vrije Universiteit Psychiatry Amsterdam Public Health research institute Amsterdam Netherlands; 2 GGZ inGeest Specialized Mental Health Care Amsterdam Netherlands; 3 Health, Sports & Welfare Cluster Nursing Inholland University of Applied Sciences Amsterdam Netherlands; 4 Amsterdam UMC, Vrije Universiteit General Practice & Elderly Care Medicine Amsterdam Public Health research institute Amsterdam Netherlands; 5 Amsterdam UMC, Vrije Universiteit Clinical Psychology Amsterdam Public Health research institute Amsterdam Netherlands; 6 Parnassia Psychiatric Institute Parnassia Academy The Hague Netherlands

**Keywords:** relapse prevention, anxiety disorder, depressive disorder, eHealth, general practice, qualitative research

## Abstract

**Background:**

Existing studies have yet to investigate the perspectives of patients and professionals concerning relapse prevention programs for patients with remitted anxiety or depressive disorders in primary care. User opinions should be considered when optimizing the use and implementation of interventions.

**Objective:**

This study aimed to evaluate the GET READY relapse prevention programs for patients with remitted anxiety or depressive disorders in general practice.

**Methods:**

Semistructured interviews (N=26) and focus group interviews (N=2) with patients and mental health professionals (MHPs) in the Netherlands were performed. Patients with remitted anxiety or depressive disorders and their MHPs who participated in the GET READY study were interviewed individually. Findings from the interviews were tested in focus group interviews with patients and MHPs. Data were analyzed using thematic analysis.

**Results:**

Participants were positive about the program because it created awareness of relapse risks. Lack of motivation, lack of recognizability, lack of support from the MHP, and symptom severity (too low or too high) appeared to be limiting factors in the use of the program. MHPs play a crucial role in motivating and supporting patients in relapse prevention. The perspectives of patients and MHPs were largely in accordance, although they had different perspectives concerning responsibilities for taking initiative.

**Conclusions:**

The implementation of the GET READY program was challenging. Guidance from MHPs should be offered for relapse prevention programs based on eHealth. Both MHPs and patients should align their expectations concerning responsibilities in advance to ensure optimal usage. Usage of blended relapse prevention programs may be further enhanced by diagnosis-specific programs and easily accessible support from MHPs.

**International Registered Report Identifier (IRRID):**

RR2-10.1186/s12888-019-2034-6

## Introduction

The high prevalence of anxiety and depressive disorders is a major public health problem, affecting 615 million people globally [1]. Although effective treatment interventions (psychological as well as pharmacological) are available [2-5], 57% of patients in remission from anxiety disorders or depression experience a relapse within 4 years [6]. Effective relapse prevention provided in general practice could increase quality of life, decrease the high burden of disease for patients with anxiety and depressive disorders, and prevent the need for treatment in a (more costly) specialized mental health care setting [7].

Existing knowledge about effective components of relapse prevention programs and effective ways of implementation remains limited. Several effective relapse prevention programs for patients with remitted depressive disorders were examined in a meta-analysis by Biesheuvel-Leliefeld et al [8], although few studies on relapse prevention concern patients with anxiety disorders [9-12]. A limited number of relapse prevention programs use eHealth, even though it offers improved access to evidence-based treatments [13]. Results concerning effectiveness in these guided eHealth studies for patients with remitted depressive disorders are conflicting: One reports a lower relapse rate after 2 years, while another does not [14,15]. Two other studies, both guided [16] and unguided [17], report a decrease in residual depressive symptoms after participating in an online relapse prevention intervention [16,17]. Variations in type of treatment, guidance, and the duration of the intervention might explain the conflicting results of these studies.

Knowledge concerning how patients and professionals value these programs is lacking. Also, knowledge regarding the appreciation of specific program components is missing. Additional insight into the valuation, feasibility, and usability of relapse prevention programs could allow optimization of such programs, as well as their implementation and use.

To our knowledge, no previous studies have investigated the perspectives of users concerning relapse prevention programs in general practice, although some do focus on users’ perspectives regarding blended interventions (eHealth combined with face-to-face contact) for depression treatment. In a study on the perspectives of patients concerning a blended cognitive behavioral therapy program for depression, Urech et al [18] reported that patients appreciate the constant availability of the online program and the possibility of reflecting on their progress. At the same time, however, patients feel pressure to complete modules, experience a lack of flexibility, and have difficulty finding motivation to complete the online modules.

In addition to the patients’ perspectives on relapse prevention interventions, it is important to consider the perspectives of the professionals providing the program: If professionals do not support the intervention, patients are less likely to use it [19]. According to professionals, advantages of blended interventions include access to online content between face-to-face sessions for patients and the fact that the structure of the online format provides focus in the treatment. At the same time, however, professionals note that technical issues could be burdensome to patients, and they do not appreciate the limited possibility of customization for online programs [20].

This article describes findings from a qualitative study conducted as part of the GET READY (Guided E-healTh for RElapse prevention in Anxiety and Depression) study [21], in which a blended relapse prevention program tailored to the patients’ preferences [22] was developed and tested. The overall aims of the GET READY study were to implement and evaluate the GET READY relapse prevention program. This program is offered by mental health professionals (MHPs) in general practices in the Netherlands to patients who are in remission from anxiety or depressive disorders.

The aim of the current study was to provide insight into the perspectives of users (both patients and MHPs) on the GET READY relapse prevention program, specifically regarding expectations of the program, attractiveness of the program, collaboration and communication between patients and MHPs, usability (especially in case of an increase of symptoms), and subjective effectiveness. In addition, the aim was to provide insight into factors that influence the use and implementation of the GET READY program in general practice.

## Methods

### Study Design

We conducted a qualitative study as part of the GET READY study. First, semistructured individual interviews were conducted with pairs of patients and MHPs. We then conducted 2 focus group interviews—one with patients and one with MHPs—to reflect on the findings from the individual qualitative interviews. The COnsolidated criteria for REporting Qualitative studies guideline [23] was followed in reporting on this study. The completed checklist can be found in Multimedia Appendix 1.

### Sampling and Recruitment for the Qualitative Study

For the individual interviews, purposive sampling was performed (based on sex, age, clinical variables, and place of residence) among all 113 patients participating in the GET READY intervention program, with the aim of including 12-15 patients and 12-15 of their MHPs. All patients enrolled in the GET READY study were at least in partial remission from an anxiety or depressive disorder and had completed treatment in specialized mental health care within the past 2 years. The researchers invited patients to participate in the individual interviews by telephone or email. The MHPs of patients agreeing to participate were invited to participate as well. In the Netherlands, most general practices employ MHPs—with professional backgrounds in community mental health, social work, or psychology—to provide mental health services [24]. Besides screening, diagnostics, providing psychoeducation, and supporting self-management, one of their tasks is to support relapse prevention [25]. Patients whose MHPs declined to participate were not interviewed. By interviewing both the patient and their MHP, we aimed to gain insight into similarities and differences in perspectives within and between these groups.

For the 2 focus groups, patients and MHPs received invitations by email. Patients and MHPs participating in the individual interview could also participate in the focus group interviews.

All participants gave written informed consent and were offered a €25 (US $30.34) gift voucher for participating in the individual or the focus group interviews. The Medical Ethical Committee of the VU University Medical Center Amsterdam judged that ethical approval was not required according to Dutch legislation. The methods of the full GET READY study are described in detail in the study protocol [21]. The GET READY program consists of 3 core components: (1) relapse psychoeducation module, (2) relapse prevention plan, and (3) weekly diary in which patients can monitor their symptoms. Furthermore, 12 optional eHealth modules are offered. All modules are focused on promoting self-management skills, by providing information, exercises, videos, and examples of fictive patients. As described, this program is offered to patients by MHPs. Patients had at least one face-to-face contact with their MHP during the GET READY study and were encouraged to visit their MHP once every 3 months, for a period of 9 months. Further details about the GET READY intervention can be found in Multimedia Appendix 2.

### Data Collection

The interviews were conducted individually with patients and MHPs by JG, EKB, and two Master’s students in medicine. All researchers had prior experience with qualitative research. Separate topic lists were developed for patients and MHPs (see Multimedia Appendix 3 and Multimedia Appendix 4), based on the aims of the study, the content of the GET READY intervention, the Consolidated Framework for Implementation Research [26], and literature on qualitative research [27,28]. In short, the topic lists contained questions regarding expectations of the program, attractiveness of the program, collaboration and communication between patients and MHPs, usability (especially in case of an increase of symptoms), and subjective effectiveness of the relapse prevention program. The interviewers did not know the patients in advance. Although they were familiar with the researchers, the MHPs were encouraged to express all comments and criticism they might have.

The interviews were conducted in the patients’ homes or the general practice location between February 2018 and February 2019. Each interview lasted about 45 minutes. Data collection and analysis occurred in an iterative process, with intermediate analyses guiding subsequent data collection [29]. Data were collected until data saturation was reached (ie, when no new themes emerged from the interviews).

After completing the individual interviews, 2 focus group interviews were conducted in June 2019 and September 2019 at the research clinic, one with patients and one with MHPs. These interviews were moderated by BM, an experienced researcher in the field of qualitative methodology. In the focus group interviews, preliminary findings from the individual interviews were presented, and input from participants was collected about their perceptions on remarkable findings in the data. Each focus group interview lasted about 90 minutes.

Individual interviews and focus group interviews were audio recorded, transcribed verbatim, and summarized. The transcripts were checked for accuracy (by reading and listening) and corrected as needed by EKB. Participants were anonymized from transcription to the reporting of the data, with only the interviewers having access to the identification key.

### Data Analysis

Data from the first sequence of individual interviews were analyzed according to the 6 steps of thematic analysis suggested by Braun and Clarke [29]. All interviews were read and re-read carefully, and initial ideas about the content of the data were recorded in the field notes (Step 1). All interviews were coded independently by 2 researchers using MAXQDA 12 [30] (EKB and JG or Master’s student). This was followed by comparing the codes and resolving disagreements through discussion. After 3 interviews, a first draft of the coding tree was prepared, and it was supplemented or adjusted regularly, based on the intermediate analyses of data (Step 2). We searched the coded data for themes, which we subsequently reviewed and defined. Preliminary themes and subthemes were discussed within the project group. Coded segments were divided among the themes and read carefully, and relevant segments were selected. A summary was prepared for each theme (Steps 3, 4, and 5). The final step consisted of producing a comprehensive and detailed report of relevant segments for each theme and selecting the most compelling ones.

## Results

### Demographic and Clinical Characteristics

Demographic and clinical variables of the patients and MHPs are presented in Table 1. Our sample contained 13 pairs of patients and their MHPs, resulting in 26 individual interviews. One MHP was interviewed twice about 2 different patients. Seven patients participated in the focus group interview. None of the patients participated in both the individual and focus group interviews. Six MHPs participated in the other focus group interview, 2 of whom had also participated in an individual interview. Reasons for nonparticipation are provided in Multimedia Appendix 5.

**Table 1:** Demographic and clinical characteristics of patients and mental health professionals (MHPs).

Characteristics		Individual interviews			Focus group interviews	
		Patients (n=13)	MHPs (n=12)	Patients (n=7)		MHPs (n=6)
Age range (years)		21-63	27-58	31-70		41-60
**Age (years), n**						
	20-39	6	4	2		0
	40-59	5	8	3		5
	≥60	2	0	2		1
**Sex, n**						
	Female	9	11	4		5
	Male	4	1	3		1
**Diagnosis (in remission), n**						
	Anxiety disorder	4	N/A^a^	2		N/A
	Depressive disorder	4	N/A	0		N/A
	Anxiety and depressive disorder	5	N/A	5		N/A

^a^N/A: not applicable.

### Overview of the Themes

Three central themes emerged from the data: “perceived value of the relapse prevention program,” “usability of the relapse prevention program,” and “need for guidance.” Each theme is considered in detail in the following paragraphs, and an overview of the themes can be found in Table 2.

**Table 2:** Overview of the main themes and subthemes and a description of their content.

Main and subthemes		Content
**Perceived value of the relapse prevention program**		
	Prior expectations	Positive expectations of patients and MHPs^a^ before using the program increased motivation for use
	Evaluation of the program	Attitudes towards the program and its (subjective) effects (eg, increased awareness of relapse risks)
	Factors inhibiting use of the program	Specific factors that reduce motivation to use the program (eg, absence of current symptoms)
Usability of the relapse prevention program		Technical aspects, attractiveness, and reflection on choices in the design of the program (eg, positive or negative views about reminders)
**Need for guidance**		
	Personal contact is essential	Added value of personal contact with MHP, prerequisite for active use of the program
	Initiating contact	Belief that the other party (ie, patient or MHP) is responsible for taking initiative

^a^MHPs: mental health professionals.

### Perceived Value of the Relapse Prevention Program

The first theme emerging from the data was the “perceived value of the relapse prevention program.” This theme was defined using 3 subthemes: (1) prior expectations, (2) evaluation of the program, and (3) factors inhibiting use of the program.

#### Prior Expectations

Prior expectations of the relapse prevention program and, by extension, motivation for its use were related to several factors, starting with the current level of symptoms experienced by patients, along with the perceived risk of relapse and the expectation that the relapse prevention program could relieve symptoms. MHPs mentioned that they noticed these factors in their patients, and patients also mentioned these factors. Patients who had current symptoms, a high perceived risk of relapse, and a belief that the program could help prevent relapse appeared to have a high motivation for active use of the program.

#### Evaluation of the Program

Many patients mentioned the importance of the relapse prevention program following recovery from anxiety or depressive disorders. They particularly appreciated the active role assigned to the patients themselves within the program, thereby encouraging them to be active participants in their process to remain well. According to the patients, the program raised awareness of relapse risks:

I find it very useful to raise my own awareness, so that I become more aware of the impact I can have, and therefore be more active in my own recovery.

34003, female, 42 years old

The focus group with patients showed that, given the diversity of modules, the relapse prevention program had relevant components for all patients. Several patients explicitly mentioned that the program provided a sense of security and stability at times when they showed signs of impending relapse.

#### Factors Inhibiting Use of the Program

Patients with few or very few symptoms believed that they would experience few, if any, benefits from participating in the program, thereby reducing their motivation to use or continue to use the program. Patients in this relatively stable situation found it more difficult to imagine the possibility of a future relapse, and they saw no immediate need to engage in active relapse prevention. On the other hand, having many symptoms could also hinder the use of the program, as a perceived lack of concentration and energy was a reason for decreased use.

According to MHPs, some patients feared that the use of the relapse prevention program could actually lead to dysregulation:

But at times I got the impression that people thought “yes, I’m doing well now” and that they were frightened that if they were to do something about their condition, they would feel less well.

MHP 39, female

### Usability of the Relapse Prevention Program

Many patients and MHPs considered the online modules inviting, due to their appealing layout and normalizing effect. They indicated that the program normalized vulnerability to relapse: Completing mental health care treatment does not mean that all the symptoms and problems have been overcome nor that aggravation of symptoms can be ruled out in the future. Several patients and MHPs found the program easy to use:

Thought it was really good. I thought it was well structured. Clear, simple to use for both the MHP and the patient.

MHP 39, female

The relapse prevention program provided MHPs with practical tools for cooperating with patients in relapse prevention. The patients were motivated by the targeted suggestions for choosing relevant modules, given their problems and needs at that time. Moreover, they felt that the content of the eHealth modules corresponded to previous treatment in specialized mental health care. Some patients appreciated receiving this information again, especially as they had forgotten some of the content.

In contrast, some patients were annoyed by the repetition of information from previous treatment. Some patients found the program’s focus on anxiety *and* depression restrictive. In their opinion, this did not enhance recognizability, particularly for those who had experienced only 1 of the 2 disorders.

One adverse aspect of the practical usability was that patients had to log on to a computer to work with the online modules. The patients suggested that it would have been easier to use an app. Patients participating in the focus group regarded the pressure caused by the program (by reminders and mandatory fields) as unpleasant and often irritating. On the other hand, the reminders in the program were sometimes also seen as a necessary “stick,” which actually helped patients to continue with the program.

Patients did not always agree with each other. While some appreciated the clarity of the eHealth program, others found navigating the online platform confusing, as they had no clear overview of the available modules. They would have preferred a more intuitive program:

I sometimes found the navigation on the site rather complicated. It wasn’t very logical.

25002, female, 40 years old

This was confirmed by patients and MHPs participating in the focus groups.

### Need for Guidance

The third theme relates to the “Need for guidance.” Both patients and MHPs considered the quality of the contact between patients and MHPs essential for effectiveness in preventing relapse.

#### Personal Contact is Essential

After patients started using the eHealth modules, patients and MHPs were encouraged to have personal contact with each other once every 3 months. Patients indicated that contact with their MHPs was particularly vital to helping them use or continue to use the eHealth modules in times of reduced stability*.*

Several patients reported having become aware of their current symptoms when preparing for their contact with their MHPs, because they knew that symptom levels would be discussed. The very prospect of the meeting seemed to increase awareness, which benefitted the focus of the conversation.

Both groups identified the combination of the eHealth program and the personal contact between patients and MHPs as a factor facilitating the use of the program. They noted that these elements complement and reinforce each other and that they would be of less value separately:

I don’t think it’s possible to do it just with eHealth. And only seeing an MHP wouldn’t work either as this is just a snapshot in time and it’s difficult to provide all the background information during that session. eHealth provides more background information, while the MHP gives practical tips.

54001, female, 30 years old

MHPs reported being happy to get patients started with eHealth modules, as this meant that the patients would have an active role in their own recovery:

It is good to be able to work with the relapse prevention program, in whatever form, in between the sessions and not just let it come down to those 30 or 40 minutes a month.

MHP 25, male

The combination of reminders from the eHealth program and the MHP provided an incentive for active use of the program. In the focus group interview with patients, it became apparent that patients receiving more support from an MHP appreciated the relapse prevention program more than patients who had received less or minimal support. The latter group indicated that they would have preferred to receive more support from the MHP in using the eHealth program.

The focus groups with patients and MHPs clearly indicated the importance of tailoring the level of support to individual patients, taking into account their coping styles and current symptom levels. The data further suggest the need to establish who will take primary responsibility when symptoms increase: Is the patient able to do this, or is active support by the MHP needed?

#### Initiating Contact

During the focus group interviews, MHPs clearly differed from patients in their task interpretations. The MHPs strongly emphasized the patient’s self-management skills, while patients expected MHPs to play a more active role if and when symptoms were to get worse. The capacity of MHPs was an impeding factor, placing limits on the active approach and support of patients. As a result, patients requiring more support were not always reached successfully:

Our consultation hour is busy enough, so if, for instance, someone doesn’t show up twice in a row for an appointment, and you have called them, then that’s it. After all, we have so many patients who can’t wait to get an appointment, so that also plays a role.

MHP 34, female

## Discussion

### Principal Findings

Both the patients and MHPs in our study were predominantly positive about the blended relapse prevention program. It created awareness of relapse risks, and users appreciated its usability and accessibility. Lack of motivation, lack of recognizability, lack of support from the MHP, and symptom severity (too low or too high) appeared to be limiting factors in the use of the program. The implementation of the program was thus challenging. Several patients and MHPs regarded the program as easy to use and clearly structured, although others referred to a lack of intuitive design and overview of modules. Patients and MHPs agreed that the combination of eHealth modules and face-to-face contact is essential. According to the respondents, the MHP plays a crucial role in motivating and supporting patients in the use of the relapse prevention program. Surprisingly, the level of agreement between MHPs and their patients was high. The paired interviews revealed no striking discrepancies within the pairs of patients and MHPs. However, the focus group interviews did reveal significant discrepancies, as MHPs assumed a certain level of self-management skills in patients, while patients articulated their limitations in this respect, expressing a desire for more direct and personalized support from their MHPs.

### Limitations

This study has several limitations. One limitation of this study is the possibility of response bias, as patients knew the objectives of the study and might have given socially desirable answers. At the start of the interviews, we emphasized our openness to all feedback, including critical comments on the program. There was also a risk of selection bias, with participants who agreed to participate in the interviews possibly having been more positive towards the program than those who did not participate. Nonetheless, both positive and negative perspectives were explicitly discussed during the interviews. Furthermore, recall bias might have occurred, given the time elapsed between completing the program and the individual interview or focus group interview for some patients (range: 0-6 months for the individual interviews and 0-12 months for the focus group interviews). We noticed that some patients tended to forget which eHealth modules they had completed and whether they had received online feedback. To reduce this bias, patients could request an overview of their completed modules and number of feedback messages to and from the MHP during the interview. In addition, patients could remain engaged after the intervention period, as they received monthly newsletters about the study and still had access to the program. The longer duration between completion of the program and the focus group interviews was caused by the fact that the focus group interviews could be prepared and conducted only after all individual interviews were conducted and analyzed.

### Comparison With Prior Work

The positive attitudes of MHPs and patients towards the program and the perceived increase in awareness of relapse risks are consistent with findings from previous research on relapse prevention for depression [31-33].

Our study revealed several factors influencing implementation, including motivation, recognizability, support received from the MHP, and symptom severity. Program use and implementation are facilitated by motivation and the perceived effectiveness of the program, as well as by the presence of current symptoms and the perceived high risk of future relapse. These findings are consistent with previous findings [34,35] demonstrating that motivation and perceived effectiveness increase adherence. On the contrary, lack of motivation impeded the use of the program, specifically in patients with few symptoms. A similar finding was suggested by Biesheuvel-Leliefeld et al [36], who may have found an indication of motivation issues among remitted patients, as they had major difficulties recruiting participants for their relapse prevention study. Another factor influencing use and implementation in our study was recognizability. This seems to correspond to findings reported by Gerhards et al [34] that patient perceptions that a program is not applicable to them act as a barrier to program usage. Our results further indicate that program implementation is determined by whether patients received support from their MHPs. This finding has also been reported in previous studies [31,33,34,37,38]. Accessible personal contact with and an active approach by the MHP appears to be the key to successful implementation of a program.

No unambiguous confirmation regarding the influence of symptom level on implementation and usage of relapse prevention programs was found in the literature. Interestingly, we found that both excessively low and excessively high perceived symptom levels hindered program use and implementation. According to a literature review on dropout from internet-based treatment, adult patients with few symptoms of any psychological disorder *and* those with more severe depressive symptoms were more likely to dropout from these treatments [39]. These findings might be generalizable to relapse prevention programs for anxiety and depressive disorders, as the setup of such internet-based treatments is similar to that of existing relapse prevention programs.

We identified different perspectives regarding responsibilities, with MHPs perceiving patients to be remitted and therefore relying on their self-management skills, while patients expected support and monitoring from their MHPs, particularly when symptoms worsened. A parallel finding is described in previous literature [40], with patients feeling that general practitioners should initiate contact, while general practitioners expect patients to contact them if needed. We found that these different expectations also exist between MHPs and patients, which has not been described before in the scientific literature.

### Implications for Practice and Research

The present study highlights the importance of guidance in eHealth-based relapse prevention for anxiety and depression. The level of guidance from and engagement of the MHP emerged as crucial factors in the success of the relapse prevention program. The self-management skills of patients and desired level of support should thus be aligned in advance, particularly in case of worsening symptoms.

Because self-management skills might differ over time, among other things depending on symptoms, it is essential to discuss and align needs over the course of the contacts, possibly enhancing implementation of relapse prevention programs based on eHealth.

Given the lack of studies specifically addressing associations between symptom levels and adherence to relapse prevention programs, further quantitative studies on this association are needed. One appropriate design could involve using Ecological Momentary Assessment [41] to assess symptom levels and log data from an eHealth platform to assess adherence.

When developing new relapse prevention interventions, attention should be paid to accessible guidance by professionals, accessibility through an app, along with a clear and intuitive, flexible structure for the eHealth component. Also, specific interventions for specific diagnoses might increase recognizability.

### Conclusions

Our findings suggest that personalized guidance from MHPs should be offered for eHealth-based relapse prevention programs, taking into account the preferences of patients and their level of self-management competencies. Both MHPs and patients should align expectations and needs in advance, as well as during the intervention, in order to increase implementation and enable optimal usage.

## Appendix

Multimedia Appendix 1 COREQ checklist.

Multimedia Appendix 2 GET READY intervention.

Multimedia Appendix 3 Topic guide interview patient.

Multimedia Appendix 4 Topic guide interview MHP.

Multimedia Appendix 5 Reasons for nonparticipation.

## Abbreviations

GET READY: Guided E-healTh for RElapse prevention in Anxiety and Depression
MHP: mental health professional
