# Supplementary material for: Evaluation of a Blended Relapse Prevention Program for Anxiety and Depression in General Practice: Qualitative Study
Source: JMIR Form Res. 2021 Feb 16;5(2):e23200. doi: 10.2196/23200 (PMC7925144; doi:10.2196/23200)
Supplement: Multimedia Appendix 1 [file formative_v5i2e23200_app1.docx]

# **Multimedia Appendix 1: COREQ checklist**

|  |  | Location in manuscript (Section, page no.) |
| --- | --- | --- |
|  |  |  |
| **Domain 1: Research team and reflexivity** |  |  |
| **Personal Characteristics** |  |  |
| 1. Interviewer/facilitator Which author/s conducted the interview or focus group? | JG, EKB, AH (acknowledgements), EL (acknowledgements), BM | Page 5 |
| 2. Credentials  What were the researcher’s credentials? E.g. PhD, MD | EKB: MSc  JG: MSc  ADTM: PhD  WDS: PhD  ORM: MD, PhD  AS: PhD  NMB: MD, PhD  BM: PhD  AH: Master student  EL: Master student | Page 1 |
| 3. Occupation  What was their occupation at the time of the study? | EKB: PhD student;  JG: psychologist and researcher;  AM: psychologist and researcher;  WS: psychotherapist and researcher;  OM: general practitioner and researcher;  AVS: Professor of Clinical Psychology;  NB: psychiatrist and researcher;  BVM: Professor of Mental Health Nursing. | Page 13 |
| 4. Gender  Was the researcher male or female? | Males and Females | Page 13 |
| 5. Experience and training What experience or training did the researcher have? | All researchers had prior experience with qualitative research. | Page 5 |
| **Relationship with participants** |  |  |
| 6. Relationship established Was a relationship established prior to study commencement? | No (patients) and yes (professionals) | Page 5 |
| 7. Participant knowledge of the interviewer  What did the participants know about the researcher? e.g. personal goals, reasons for doing the research | Participants were briefed about the purpose of the study | Multimedia Appendix 3 & 4 |
| 8. Interviewer characteristics  What characteristics were reported about the interviewer/facilitator? e.g. Bias, assumptions, reasons and interests in the research topic | None |  |
| **Domain 2: study design** |  |  |
| **Theoretical framework** |  |  |
| 9. Methodological orientation and Theory What methodological orientation was stated to underpin the study? e.g. grounded theory, discourse analysis, ethnography, phenomenology, content analysis | Topic lists based on the Consolidated Framework for Implementation Research. Analyses conducted with thematic analysis. | Page 5-6 |
| 10. Sampling  How were participants selected? e.g.  purposive, convenience, consecutive, snowball | Purposive | Page 4 |
| 11. Method of approach How were participants approached? e.g. face-to-face, telephone, mail, email | Telephone or email | Page 4 |
| 12. Sample size  How many participants were in the study? | 36 participants | Page 6 |
| 13. Non-participation How many people refused to participate or dropped out? Reasons? | See Multimedia Appendix 5 | Multimedia Appendix 5 |
| **Setting** |  |  |
| 14. Setting of data collection  Where was the data collected? e.g. home, clinic, workplace | Individual interviews: patients’ homes, general practice location; Focus-group interview: research clinic | Page 5 |
| 15. Presence of non-participants  Was anyone else present besides the participants and researchers? | No |  |
| 16. Description of sample What are the important characteristics of the sample? e.g. demographic data, date | See Table 1 | Page 6 |
| **Data collection** |  |  |
| 17. Interview guide  Were questions, prompts, guides provided by the authors? Was it pilot tested? | Yes | Page 5 |
| 18. Repeat interviews Were repeat interviews carried out? If yes, how many? | Yes, one MHP was interviewed twice, since two of his patients both agreed to participate | Page 6 |
| 19. Audio/visual recording Did the research use audio or visual recording to collect the data? | Individual interviews and focus-group interviews were audio recorded, transcribed verbatim and summarized. | Page 5 |
| 20. Field notes  Were field notes made during and/or after the interview or focus group? | Yes | Page 5 |
| 21. Duration  What was the duration of the interviews or focus group? | Individual interviews: 45 minutes  Focus-group interviews: 90 minutes | Page 5 |
| 22. Data saturation  Was data saturation discussed? | Yes | Page 5 |
| 23. Transcripts returned Were transcripts returned to participants for comment and/or correction? | No |  |
| **Domain 3: analysis and findings** |  |  |
| **Data analysis** |  |  |
| 24. Number of data coders How many data coders coded the data? | 4 | Page 5 |
| 25. Description of the coding tree  Did authors provide a description of the coding tree? | Yes | Page 6 |
| 26. Derivation of themes Were themes identified in advance or derived from the data? | Themes were derived from the data. | Page 5-6 |
| 27. Software  What software, if applicable, was used to manage the data? | MAXQDA 12 | Page 5 |
| 28. Participant checking Did participants provide feedback on the findings? | Yes, by performing the focus-group interviews. | Page 5 |
| **Reporting** |  |  |
| 29. Quotations presented Were participant quotations presented to illustrate the themes / findings? Was each quotation identified? e.g. participant number | Yes | Page 8-10 |
| 30. Data and findings consistent  Was there consistency between the data presented and the findings? | Yes |  |
| 31. Clarity of major themes  Were major themes clearly presented in the findings? | Yes | Page 7-10 |
| 32. Clarity of minor themes  Is there a description of diverse cases or discussion of minor themes? | Yes | Page 7-10 |
