# Supplementary material for: Evaluation of a Blended Relapse Prevention Program for Anxiety and Depression in General Practice: Qualitative Study
Source: JMIR Form Res. 2021 Feb 16;5(2):e23200. doi: 10.2196/23200 (PMC7925144; doi:10.2196/23200)

# **Multimedia Appendix 2: GET READY intervention**

The GET READY relapse-prevention program was developed for general practice and consists of several elements. First, patients are invited by their MHPs for a face-to-face session (F2F), in which an individual relapse-prevention plan is discussed. Second, patients receive access to an E-health platform. Third, patients are monitored by their MHPs and can schedule regular F2F sessions. The E-health platform provides three basic components and 12 optional modules, which patients can select and prioritize according to their needs and preferences (Figure 1). Each module takes 20-30 minutes to complete. Based on module completion, pop-up suggestions appear about related modules that might be interesting to the patient. A diary is also available, in which patients monitor symptoms weekly. The diary is available via the computer and via an app. All other modules are accessible through the computer. All modules aim to promote self-management skills (e.g. seeking help in case of impending relapse or pro-active planning of healthy behavior to cultivate stability and psychological and physical wellbeing). Patients receive weekly reminders to complete the selected online modules, receiving feedback from their MHPs upon request.

Overview of E-health modules, adapted from previous publication [20]. Dotted lines indicate that modules have overlapping themes and that modules can be easily opened from the other modules.


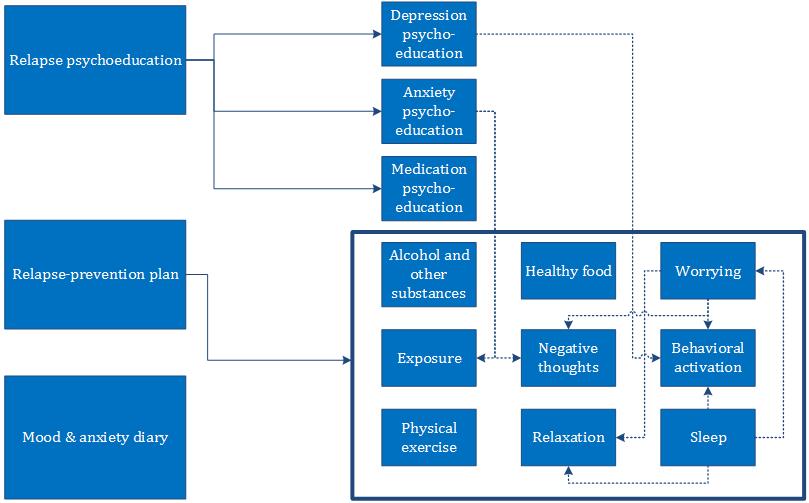

Supplement: Multimedia Appendix 2 [file formative_v5i2e23200_app2.docx]
