# Supplementary material for: Evaluation of a Blended Relapse Prevention Program for Anxiety and Depression in General Practice: Qualitative Study
Source: JMIR Form Res. 2021 Feb 16;5(2):e23200. doi: 10.2196/23200 (PMC7925144; doi:10.2196/23200)
Supplement: Multimedia Appendix 3 [file formative_v5i2e23200_app3.docx]

# **Multimedia Appendix 3: Topic guide interview patient**

| **Main questions** | **Additional questions** |
| --- | --- |
| Introduction | Sign informed consent  Introduce yourself  Mention the duration of the interview (45 minutes)  Ask permission to audio record the interview, and start the recording  Mention that the name and personal data is saved separately from research data  Mention the purpose of the interview |
| What is your experience with the relapse prevention program in general? | What were your expectations of the relapse prevention program?  Could you tell more about the role of the MHP?   - Did you already know the MHP at the beginning of the study? - What did you expect from the MHP at the beginning of the study? - How did you experience the contacts? - How did you experience the support from the MHP? - In which way did the MHP help you to stay healthy? - What could be improved in the guidance from the MHP? - Who initiated the contact and how did you experience this? - What did you expect from the MHP if symptoms worsened?   What is your experience with the E-health program?   - Usability - Meeting your needs - Pleasure/satisfaction - Available choices - Use of language - Design - Time investment   Did the program help you to stay healthy/without symptoms?   - In which way did the program help you to stay healthy? - If the program did not help: did other things help? - If you experienced a period of worsening symptoms: what would you have needed to use the program?   How do you feel about the combination of E-health and having contact with the MHP?   - If you had contacts with the MHP but did not use E-health: what motivated you to have contact with the MHP? |
| What were the most useful and the least useful parts of the E-health program? | - Which modules did you complete? What made you choose these modules? - What were your motives concerning using or not using the modules? - Which modules were set up for you, but not completed? - Did you complete the diary? - How much did you use the message function within the program? - According to the questionnaires, your symptoms have decreased/increased. How did you experience this? |
| What do you like about the relapse prevention program and what could be improved? | What is the most useful aspect of the relapse prevention program?  What could be improved in the relapse prevention program?  What did you miss in the relapse prevention program? |
| Completion | Are there other topics you would like to discuss?  Do you have any questions?  Would you like to receive the outcomes of the study?  Would you be interested in participating in a focus group interview? |
