# Supplementary material for: Evaluation of a Blended Relapse Prevention Program for Anxiety and Depression in General Practice: Qualitative Study
Source: JMIR Form Res. 2021 Feb 16;5(2):e23200. doi: 10.2196/23200 (PMC7925144; doi:10.2196/23200)
Supplement: Multimedia Appendix 4 [file formative_v5i2e23200_app4.docx]

# **Multimedia Appendix 4: Topic guide interview MHP**

| **Main questions** | **Additional questions** |
| --- | --- |
| Introduction | Sign informed consent  Introduce yourself  Explain which patient the interview is about  Mention the duration of the interview (45 minutes)  Ask permission to audio record the interview, and start the recording  Mention that the name and personal data is saved separately from research data  Mention the purpose of the interview |
| Practical questions | How many hours a week do you work as MHP?  Do you work in multiple general practices?  How many minutes does a regular contact last?  How many MHPs/GPs are working in this general practice? |
| What is your experience with the relapse prevention program? | How did you offer the relapse prevention program to the patient?   - How were follow-up contacts planned? - Who initiated the contact and how did you experience this? - How many contacts did you have? - What did you discuss during the contacts? - Did you already know the patient before the beginning of the study? - Did you stimulate the patient to use the relapse prevention program? In which way? - How did the program influence the health of the patient? - To what extent did the program meet the patients’ symptoms?   How did you offer the relapse prevention program to other patients?  *What was it like to offer the relapse prevention program to the patient?  What is your experience with the E-health program?   - Usability/structure - Design - Use of language - Time investment - Aspects: what did you use/not use, experience with aspects, relapse prevention plan - Message function/providing feedback |
| What made it easier or harder to implement the relapse prevention program in the general practice? | The relapse prevention program itself: intervention characteristics   - What did you think about the quality of the relapse prevention program? - Did you know the content of the modules? - Did you ever apply relapse prevention strategies before? How does this program compare to other strategies that you are familiar with? - How do you feel about the combination of contacts and E-health? - *How complicated is the intervention?   Factors within the general practice: inner setting   - How could you apply the relapse prevention program in your ‘normal work’? - Did you experience support from the general practice or GP? How? - In the general practice, what is the willingness to change? - How did having/not having time affect implementation?   Characteristics of individuals   - Did you feel confident offering the relapse prevention program? Why/why not?   Process of implementation   - Did you experience support from the research team? How?   Influence from outer setting   - Did you have contact with specialized mental healthcare services? What was the effect? |
| What do you like about the relapse prevention program and what could be improved? | What is the most useful aspect of the relapse prevention program?  What could be improved in the relapse prevention program?  *What did you miss in the relapse prevention program?  Would you use the relapse prevention program if available after completion of the study? What is needed? |
| Completion | Are there other topics you would like to discuss?  Do you have any questions?  Would you like to receive the outcomes of the study?  Would you be interested in participating in a focus group interview? |

* If not discussed yet, also ask this question
