# Supplementary material for: Evaluation of a Blended Relapse Prevention Program for Anxiety and Depression in General Practice: Qualitative Study
Source: JMIR Form Res. 2021 Feb 16;5(2):e23200. doi: 10.2196/23200 (PMC7925144; doi:10.2196/23200)
Supplement: Multimedia Appendix 5 [file formative_v5i2e23200_app5.docx]

# **Multimedia Appendix 5: Reasons for non-participation**

Initially, 35 patients were invited for the individual interviews. Of the 19 agreeing to participate, 13 (37%) were selected and interviewed. In all, 17 MHPs were invited for the individual interviews. Of the 14 agreeing to participate, 12 (71%) were selected and interviewed. Reasons for non-participation are presented in Table 1.

Table 1. Reasons for non-participation in individual interviews

|  | Invited | Agreed to participate | Interviewed | Reasons for non-participation |
| --- | --- | --- | --- | --- |
|  |  |  |  |  |
| **Patients** | 35 | 19 | 13 | Demographic variables overlapped with participating patients (N=6) First agreed to participate but later unreachable (N=6) Too difficult to talk about (N=2)  Comorbid symptoms (N=2)  MHPs did not wish to participate (leading to non-participation by patients) (N=2)  No interest (N=2)  No time (N=1)  Did not use the program (N=1) |
| **MHPs** | 17 | 14 | 12 | No interest (N=2) Patient did not wish to participant (leading to non-participation by the MHP) (N=1) Already interviewed about another patient (N=1) Not reachable (N=1) |

In all, 98 patients were asked to participate, and seven (7%) participated in the focus-group interview. Of the 50 MHPs invited to participate, six (12%) participated in the focus-group interview. Reasons for non-participation are presented in Table 2.

Table 2. Reasons for non-participation in the focus-group interview

|  | Invited | Interviewed | Reasons for non-participation |
| --- | --- | --- | --- |
|  |  |  |  |
| **Patients** | 98 | 7 | No contact (N=55)  No interest (N=16)  No time (N=16)  Not able to travel (N=2)  Did not use the program (N=1)  Too difficult to talk about (N=1) |
| **MHPs** | 50 | 6 | No contact (N=39)  No interest (N=4)  No time (N=1) |
